# Preparation and Tribological Study of Graphene Coating on Glass Fiber-Reinforced Composite Using Modified Percolating-Assisted Resin Film Infusion Method

**DOI:** 10.3390/ma13040851

**Published:** 2020-02-13

**Authors:** Ben Wang, Wei Han, Yueke Ming, Xiaohui Zhang, Yansong Zhu, Yugang Duan, Hongxiao Wang, Hongying Zhao

**Affiliations:** 1 State Key Lab for Manufacturing Systems Engineering, Xi’an Jiaotong University, Xi’an 710049, China; wangben@xjtu.edu.cn (B.W.); jx18hanwei@stu.xjtu.edu.cn (W.H.); mingyueke@foxmail.com (Y.M.); xjtu_zys@stu.xjtu.edu.cn (Y.Z.); xawben@gmail.com (Y.D.); 2 Polymer materials and plastics technology, Clausthal University of Technology, 38678 Clausthal-Zellerfeld, Germany; hongying.zhao@tu-clausthal.de; 3 College of Mechanical and Electrical Engineering, Henan university of Technology, Zhengzhou 450001, China; xawhx2013@xjtu.edi.cn

**Keywords:** friction mechanism, graphene, fiber-reinforced composites, wear resistant

## Abstract

Tribological properties of glass fiber-reinforced polymer (GFRP) composites used in reciprocating contact should be improved to secure the efficiency and safety because of risks of abrasion, adhesion, and fatigue deficiency amidst fiber, matrix, or interphase. This paper investigates the influence of graphene reinforcement on the wear resistance of a GFRP composite. Graphene was integrated into a typical GFRP composite as the surface coating using a modified resin film infusion method with the percolating paper assisted. Dry reciprocating sliding tests were performed against a stainless steel ball moving in a direction 45 degrees to the fiber orientation. The morphology of the worn surface was observed, and the corresponding wear mechanisms are discussed. Results suggest that the prepared graphene coating improves the wear resistance of the GFRP composite. The protected GFRP laminates remained intact during the first 20 min of the wear test and only a small fraction of fibers were broken after 60 min test. Furthermore, abrasive debris and fiber breaks originating from composite were markedly reduced, likely owing to the formation of a protective transfer film between the surface of the modified composite and the rubbing counterpart.

## 1. Introduction

Glass fiber-reinforced polymer (GFRP) composites are one of the most rapidly growing classes of material, owing to the combination of high specific strength and high specific modulus. Nowadays, GFRP composites are widely used in a variety of engineering applications, such as pipes in the petroleum and mining industries, helicopter rotor blades, pump impeller blades, high-speed vehicles, and aircraft parts [1,2]. Although glass fiber reinforcement offers better mechanical and thermal performance, poor chemical activity of fiber surface contributes to deteriorate the interphase of composite in the sliding abrasion process, which leads to the insufficient tribological performance. Moreover, according to a recent tribological survey, abrasive wear is responsible for the largest amount of material loss in industrial applications [3]. Friction can cause brittle glass fibers to rupture, leading to debris on the worn surface. Owing to the insufficient lubrication, these hard debris act as abrasives between the two surfaces arousing abrasive wear, as a result, aggravating the wear friction. In order to reduce the abrasion failures, two primary popular approaches are normally adopted to improve the friction reduction and the antiwear abilities of materials. Lubricant materials, such as polytetrafluoroethylene [4], graphite flakes [5], and boron carbide [6], can significantly improve the wear performance by forming a strongly adhering and tenacious, yet lubricating, transfer film on the surface of the composite material, thereby providing improved wear resistance against the rubbing counterpart [7]. The second approach aims to improve the strength and durability of the resin matrix or interphase of the GFRP so that it can withstand impacts from the counterpart and reduce abrasive damage of the composite. To this end, several methods have been proposed. Nanoparticles provide effective reinforcement for composites and can improve their tribological properties. Carbon nanofillers (e.g., carbon nanotubes [8] and carbon nanofibers [9]) and ceramic nanofillers (e.g., aluminum oxide (Al_2_O_3_) [10], silicon carbide (SiC) [11], and nanosilica (SO_2_) [7]) can be incorporated to strengthen the mechanical properties of the resin matrix, with a minimal increase in weight [12]. 

Among the available fillers and reinforcements, graphene can endow composites with excellent self-lubricating properties by forming graphitic structures on the surface of the material [13]. In addition, graphene also exhibits remarkable reinforcing performance owing to its high modulus and high strength, along with an extremely large surface area [14]. In terms of the lubricating effect, graphene can suppress wear since its in-plane dimensions are in the order of several microns coupled with a nanometer-scale sheet thickness. Graphene adheres to the composite surface forming a low-friction film, thus, preventing direct contact between the composite material and rubbing counterpart. For example, graphene was shown to form a transfer film on nylon composites, and successfully reduced wear and improved friction performance under dry sliding [15]. Moreover, the lower friction coefficient of graphene-filled Nomex fabric composites can be attributed to the graphene transfer film that is easily formed on the surface of the composite [16]. In terms of reinforcement, the additional stiffness of graphene embedded in the composite matrix can prevent debris formation. For example, the wear rate of Polytetrafluoroethylene (PTFE) was dramatically reduced from 0.4 × 10^−3^ mm^3^/Nm to 10^−7^ mm^3^ after incorporating graphene, which improved the fracture toughness and fatigue performance [17]. Graphene can also enhance interphase adhesion/interlocking and the glass transition temperature of composites, thus this reinforcing effect further improves the wear resistance of the composite [18].

Based on the discussion above, graphene has been shown to improve antifriction and antiwear performance synchronously. However, further improvements of wear performance by combining both the lubricating and reinforcing effects of graphene have not been evaluated. Furthermore, processability of composite structures remains an important issue to address. For instance, buckypaper and other films are not large enough to cover the surface of large-scale composite structures, such as turbine blades or the skin of an aircraft, mainly owing to the limitations of current manufacturing techniques [19]. Also, the viscosity of the resin matrix can dramatically increase when graphene is introduced, which can negatively affect existing manufacturing processes for composite materials [20].

To overcome these issues, this paper investigated the influence of graphene reinforcement on the wear resistance of a GFRP composite. Different forms of graphene (a coating preparation and embedded in interlayers) were compared in terms of their antiwear performance. To realize the designed structure, a modified percolating-assisted resin film infusion method was used to add an equivalent amount of graphene to preformed glass fiber-reinforced laminate. Finally, the results of dry reciprocating sliding tests were used to compare the effectiveness of each preparation.

## 2. Experimental

### 2.1. Materials

The epoxy matrix system used in this study was comprised of bisphenol A epoxy resin (EPOLAM 5015, Axson, Saint ouen l’aumone, France), phenolic epoxy resin (Institute of Aeronautical Materials, Beijing, China), and polyethersulfone (PES, Changchun Jilin University Special Plastic Engineering, Changchun, China). The 4,4′-diamino-diphenylsulfone (DDS, Shanghai Experiment Reagent, China) was used as the curing agent. Large-diameter graphene with a length of 7–12 μm and 1–3 layers was obtained from Guosen Shenzhen company. The graphene was produced using a mechanical exfoliation method to maintain its stiffness and strength [21]. Bidirectional, plain woven high-strength glass fabric was used as the fiber reinforcement (areal weight of 110 g/m^2^, CW200XP, Taishan Fiberglass Inc. Taishan, China). All vacuum bagging materials were supplied by Sino-composite Co. Ltd. (Beijing, China)

### 2.2. Composites Fabrication 

**Figure 1:**
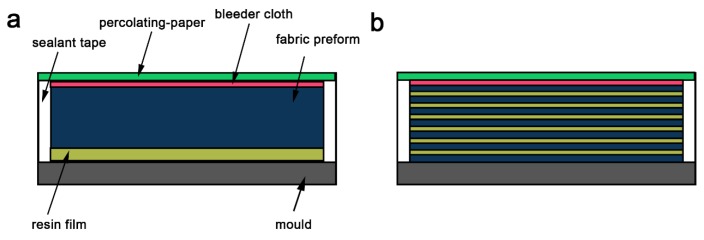
Schematic illustration of the preform fabrication process using percolating-assisted resin film infusion method: (**a**) Coating preparation, (**b**) embedded interlayer preparation.

The multi-scale reinforced laminate was fabricated using percolating-assisted resin film infusion, according to a previously described protocol [19]. Briefly, a specific amount of graphene was first dispersed in the resin matrix using a high-energy ball milling method, followed by desolventizing the solvent. Then, the PES film-forming agent and curing agent were added one by one and mixed at 120 °C. The resulting solution was cast on a hot plate using the flow-casting method to obtain the resin film with graphene dispersion [19]. To compare the effectiveness, an equivalent amount of graphene (e.g., 0.4 g) was adopted for the coating and the embedded interlayer preparation. The resulting solution was cast averagely into 7 pieces of resin film for the embedded interlayer preparation, in order to disperse evenly the graphene addition in each monolayer. An entire resin film containing the total graphene addition was used as the coating preparation. The typical process for producing the resin film is shown in Figure 1a. Briefly, in terms of coating preparation, as-prepared nano-hybrid resin film (120 mm × 120 mm) was placed on a heated mold, then glass fabric, percolating paper (pore size of 1–4 μm), and bleeder cloth were added in sequence. Analogously, 7 pieces of nano-hybrid resin film were placed between each glass fabric as the interlayer molding (Figure 1b). The edges of the preformed fabric were tightly sealed with sealant tape as dams to ensure resin flow in the thickness direction. After that, the arrangement was sealed with a vacuum bag and sealing tape. The entire infusion molding process was conducted according to a previously described protocol [19]. Briefly, the preformed fabric was heated to 120 °C for 3 h until the resin film completely melted and infused between the fabric piles. A vacuum pressure was applied to extract excess resin from the bleeder cloth. During the impregnation, the percolating paper kept the embedded graphene between the preformed fabric. Finally, the prepared laminate was postcured in an autoclave for 4 h at 180 °C and then for 2 h at 200 °C. Prepared samples were labeled as C-n for the coating preparation, and I-n for interlayer embedment, where n denotes the weight of graphene.

### 2.3. Characterization of Prepared Composites

The prepared coating samples were observed by digital optical camera (VH-8000, Keyence, Osaka, Japan) and scanning electron microscopy (SEM, S–3000N, Hitachi, Tokyo, Japan). The cross-sections of samples were prepared by grinding and polishing before the observation. Reciprocating sliding tests were performed to investigate the wear performance and the coefficient of friction (COF) of the composite. Prepared samples were tested on a multifunction wear tester (CFT-1, Lanzhou Zhongke Kaihua Technology Development Co., Ltd. Lanzhou, China) and a stainless steel ball (GCr15, diameter of 5 mm) with a hardened and smoothly polished surface served as the counterpart. All samples were degreased and cleaned using acetone and ethanol before testing. Reciprocating tests were carried out at 23 °C in ambient air and testing cycles were implemented for 20 min and 60 min. A normal load of 30 N (Newton) was applied to the sample at a speed of 300 rpm and the total moving distance was 5 mm. To accelerate the wear behavior and obtain a relatively even surface morphology, prepared samples were fixed using a jig with an angle of 45° between bidirectional fibers and the sliding direction [22]. The wear morphology of the tested samples was analyzed by scanning electron microscopy and confocal optical microscopy (OLS4000, Olympus, Tokyo, Japan). In detail, the confocal optical microscopy in an optical observation model was employed to inspect the worn surfaces after the tests, while 3D surface topography of the worn surfaces was examined in a confocal laser scanning model. Due to the observation dimension limitation of optical microscopy (2.5 × 2.5 mm^2^), the observations of the worn surface were focused on the middle section of the whole abrasion area that contained the deepest point in the worn surface, in order to give a representative damage of the worn morphology.

## 3. Results and Discussion

### 3.1. Preparation Characterizations

**Figure 2:**
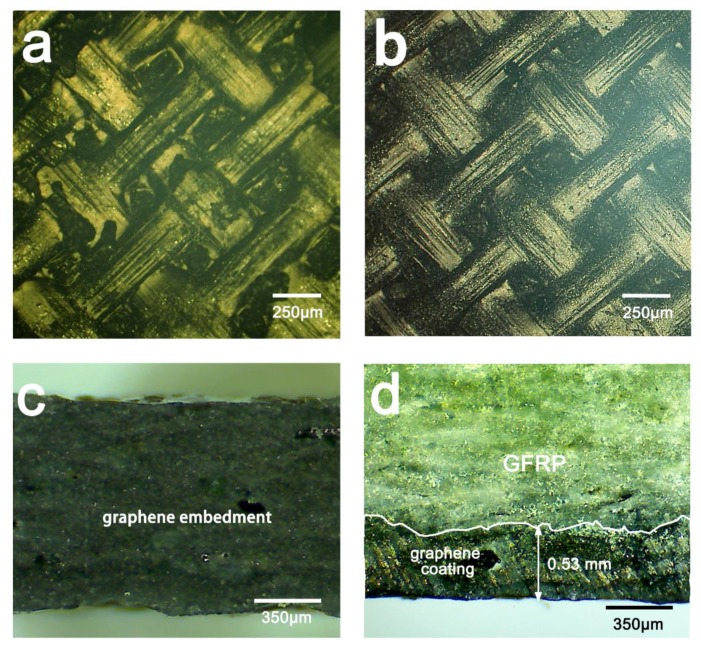
Optical images of glass fiber-reinforced polymer (GFRP) composite laminate with graphene preparation: (**a**,**b**) Surface morphology and (**c**,**d**) cross-section of samples with graphene embedded in interlayer (**a**,**c**) and coating (**b**,**d**).

Identical amounts of graphene (0.4 g) were introduced into GFRP laminates by either interlayer embedment or coating preparation. Surface morphology of prepared samples are shown in Figure 2a,b. Compared to interlayer embedment, more graphene was embedded in the laminate surface using coating preparation. Typically, graphene was distributed on the surface of the laminate and trapped in overlapping fibers. A representative cross-section of samples prepared using interlayer embedment showed an intact dark section, indicating that graphene penetrated the entire thickness of the laminate and was dispersed throughout the interlayer (Figure 2c). In contrast, a representative cross-section of the coating preparation showed only a thin black layer of around 0.53 mm on the surface indicating embedded graphene, whereas the rest of the sample exhibited a pristine GFRP structure (Figure 2d). 

**Figure 3:**
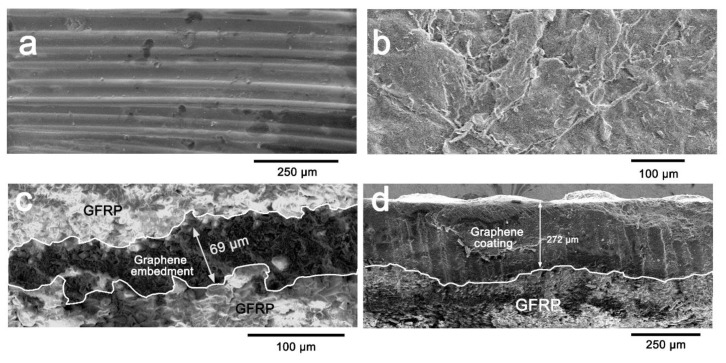
Representative SEM images of GFRP composite laminate with graphene preparation: (**a**,**b**) Surface morphology and (**c**,**d**) cross-sections of samples with graphene embedded in interlayer (**a**,**c**) and coating (**b**,**d**).

**Figure 4:**
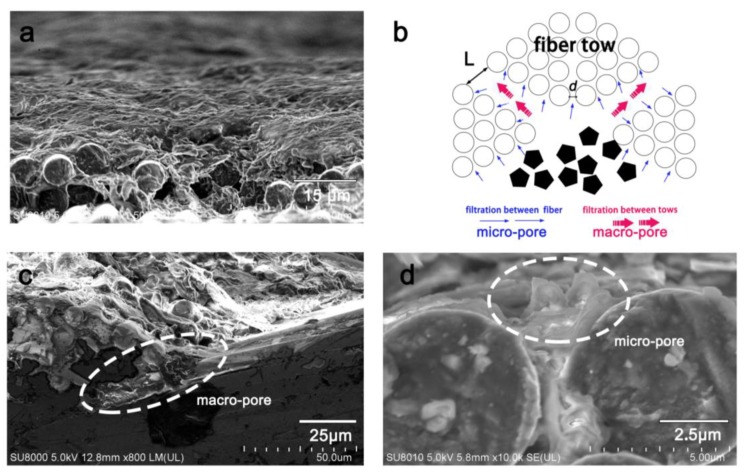
Schematic explanation of the graphene coating preparation: (**a**) C-0.005 g, (**b**) illustrations of graphene flow in the fibrous fabric, magnified images of macropore (**c**) and micropore (**d**) in the coating preparation.

Enlarged images of the prepared samples (N-0.4 g and I-0.4 g) are presented in Figure 3. Fibers can be clearly observed on the surface of samples produced by interlayer embedment (Figure 3a). Moreover, very little graphene can be observed on the surface. A cross-sectional view (Figure 3c) shows a darker layer of embedded graphene between layers, indicating graphene was constrained between the fibrous fabrics. In contrast, the surface fibrous fabrics were covered with the graphene coating (Figure 3b). Graphene accumulated during the molding process, leaving an integrated coating to a depth of nearly 272 μm. Compared to Figure 2d, the entire graphene region in the sample exceeded that of the surface graphene coating, suggesting that some graphene infused in the fibrous medium during the resin infusion process. In Figure 4a, a graphene coating barely formed in C-0.005 g, suggesting graphene first permeated the fibrous fabric, and then accumulated on the surface. Based on our previous study [19], two available channels were assigned to impede the movement of graphene in the resin flow (Figure 4b). The first can be attributed to macropores between the fiber tows (Figure 4c), which mostly influence resin flow. Graphene fillers were first crushed in these channels as the resin film began to fuse with the fibrous medium. However, the flow channel of macropore gradually became clogged with continuously captured graphene, and the macrofiltration process will be quickly transformed into microfiltration via the inner channels between filaments (Figure 4d). Finally, all available flow channels or pores became blocked with graphene and, as a result, redundant graphene accumulated, forming a graphene coating on the surface of the fibrous fabric. 

**Figure 5:**
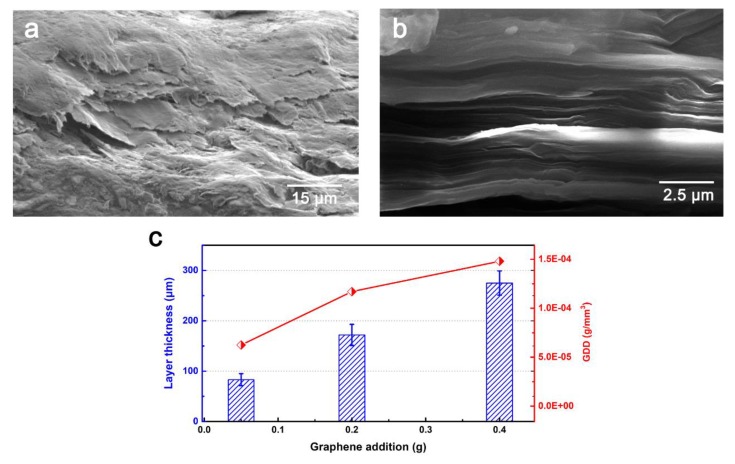
(**a**) Accumulation of graphene in coating preparation. (**b**) Typical appearance of graphene buckypaper prepared by vacuum filtration method. (**c**) Coating thickness and graphene distribution density (GDD) in coatings with different amounts of graphene.

In Figure 5a, two-dimensional graphene platelets with an extremely high aspect ratio preferentially oriented parallel to each other in the prepared coating, resembling the structure of graphene buckypaper prepared using the vacuum filtration method (Figure 5b) [23]. It implies graphene coating by our proposed method can be explained by the vacuum filtration mechanism. When graphene dispersed in the resin flow was subjected to vacuum filtration, the fibrous fabric acted as the filter medium, thus leading to assemble graphene packed and horizontally aligned bulk. In addition, increased graphene resulted in a thicker coating, however, the graphene density distribution (GDD, i.e., graphene weight in per unit volume of coating) exhibited a rising trend, suggesting the graphene structure became denser as it continued to accumulate during the molding process. The possible mechanism will be investigated in the future. For now, it can be concluded that more graphene results in a denser coating, which should improve wear resistance.

### 3.2. Tribological Performance

**Figure 6:**
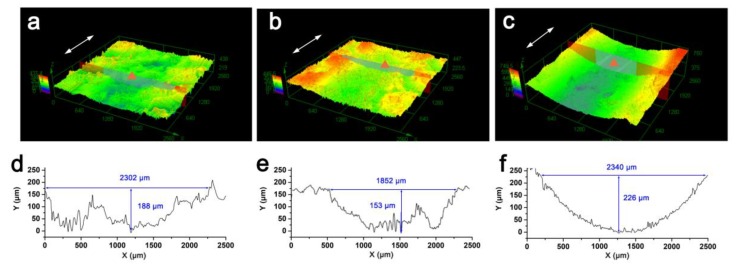
Surface features of composites: (**a**–**c**) Three-dimensional surface topography and (**d**–**f**) surface profiles of samples with raw GFRP (**a**,**d**), I-0.4g (**b**,**e**), and C-0.4 g (**c**,**f**).

Worn surface after the first 20 min of the friction test were presented (Figure 6a–c). The interlayer embedment (I-0.4 g) resulted in the least amount of wear damage, whereas the cambered worn surface of the coating sample (C-0.4 g) suggested more severe wear, even compared with raw GFRP. Moreover, the morphology of the worn surface of the graphene embedment sample was smooth with only alleviated damage. After the test, the surface of the coating sample was circular in shape and scratches and pits were difficult to identify (Figure 6c), suggesting lower roughness. 

In contrast, the raw GFRP and the interlayer embedment samples exhibited a bumpy, worn surface. The worn surface profiles at the deepest point (marked as the triangle symbol in Figure 6a–c) are illustrated in Figure 6d–f. Generally, damage to the interlayer embedment sample was less deep and less spread out compared to the raw GFRP, although damage to the coating sample (both worn depth and width) was less significant than damage to the interlayer embedment sample. For the coating sample, the depth of wear exceeded 226 μm, suggesting that sliding wear did not penetrate the layer integrated coating (intact depth of 272 μm). That is, the inner GFRP composite remained intact during the wear test. 

**Figure 7:**
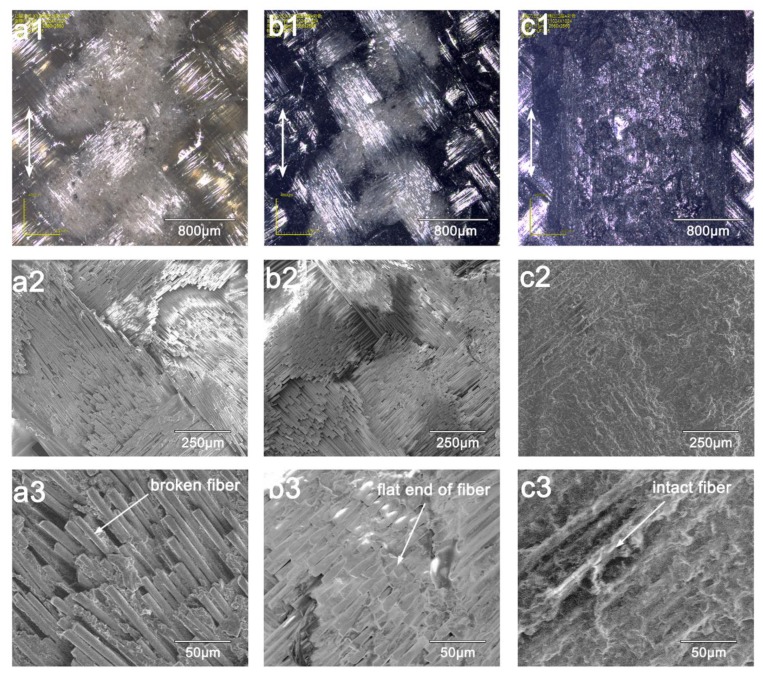
SEM images of worn surfaces of composites: (**a**) Raw GFRP, (**b**) I-0.4 g, (**c**) C-0.4 g.

SEM images of the worn surfaces for raw GFRP and graphene-modified composites are shown in Figure 7. For the raw GFRP, fibers and resin at the center of the worn region were completely shelled off. Moreover, fiber breakage on the surface caused the rest of the worn region to appear blurred (Figure 7a1). High repeated shear stress acting on the fibers during the sliding movement of the counterpart resulted in fiber damage and pull out of fibers, as shown in Figure 7a2. Also, some of the remaining resin covered the fibers (Figure 7a3), indicating that the resin matrix was worn away due to poor bonding adhesion between glass fibers and matrix [24]. 

When graphene was introduced in the interlayer, some of the damage was alleviated (Figure 7b1). In addition to reducing the width of the worn area, the region of fiber breakage became smaller. Although fiber breakage did take place at the center of the worn region, most of the remaining resin was still wrapped around the fibers (Figure 7b2). Moreover, broken ends of fibers on the worn surface of the raw GFRP were flat and brittle, which would elicit a grinding effect on the surface of the interlayer embedment sample (Figure 7b3). 

The plasticity limit of the glass fiber was reached during sliding movement, contributing to brittle fracture [25]. However, the presence of graphene in the resin matrix can improve the bonding strength between fiber and matrix, allowing broken fibers in the matrix to endure the sliding wear for longer. A larger worn surface is shown in Figure 7c1, suggesting that the graphene coating layer was seriously worn after the test. However, a magnified image shows fibers were still secured by the graphene layer, and, consequently, no fiber breakage can be observed in Figure 7c2,c3. 

**Figure 8:**
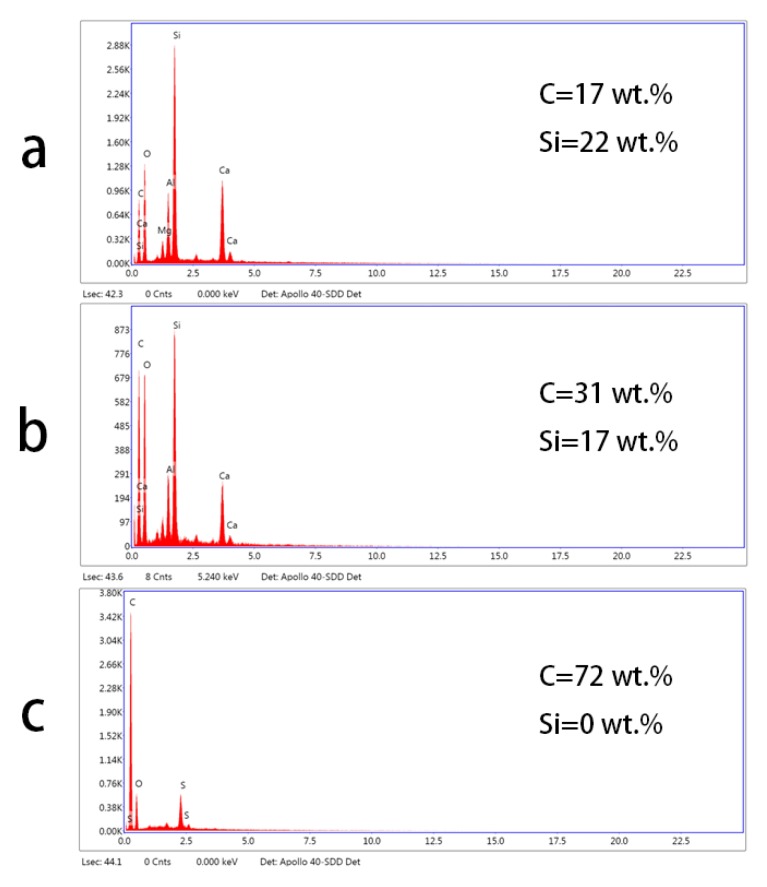
EDS analysis of worn surface: (**a**) Raw GFRP, (**b**) I-0.4 g, (**c**) C-0.4 g.

Energy-dispersive X-ray spectroscopy (EDS) characterization of the center of the worn surface can further explain the friction behavior (Figure 8). The worn surface of the interlayer embedment sample possessed a similar chemical composition compared to raw GFRP, including silicon, calcium, and aluminum elements, indicating glass fibers were present (Figure 8a,b). This also suggests that glass fiber debris was directly exposed to the counterpart. However, when the graphene coating was used on the surface, the majority of elements on the surface were indicative of carbon, suggesting the protective graphene layer served as the transfer film between the GFRP and the counterpart (Figure 8c). Therefore, even if a weak GFRP interface causes glass fibers to detach from the resin, graphene on the surface can withstand the sliding abrasion process and protect fibers from breakdown [16,26]. As a result, samples with the graphene coating only exhibited mild damage and the detachment of glass fiber was prevented. 

**Figure 9:**
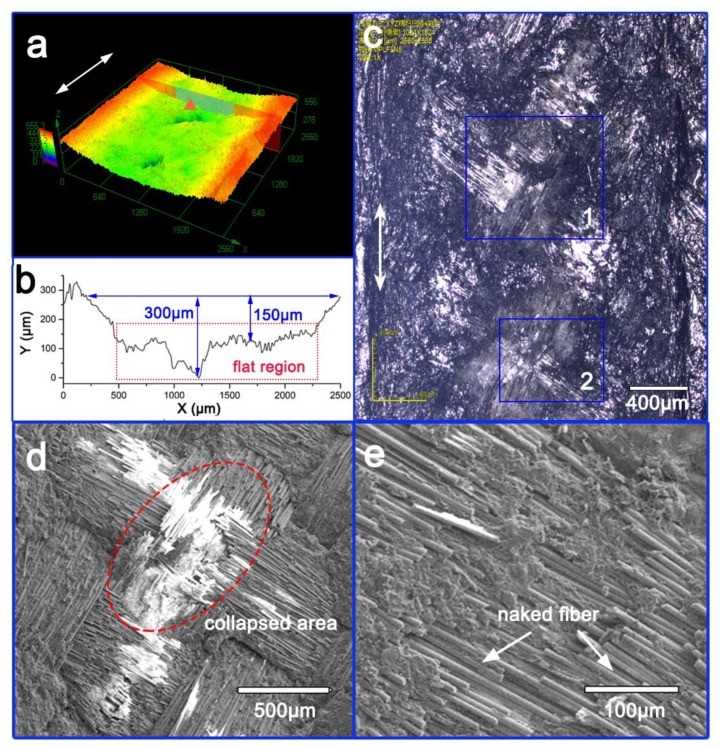
Morphology of worn surface of B-0.4 g: (**a**) 3D surface topography, (**b**) surface profile, (**c**) optical image of worn surface, (**d,e**) enlarged SEM images of Sections 1 and 2 from Figure 8c.

To further study the antiwear resistance of the integrated surface coating, a typical buckypaper of graphene was selected as a surface coating and a comparative analysis was performed. The buckypaper coating containing an equivalent amount of graphene (0.4 g) was prepared using a previously described protocol [23], and co-cured with the GFRP preform, labeled as B-0.4 g. The obtained samples possessed a thickness of around 150 μm. After the 20-min wear test, the 3D surface topography was observed. The surface displayed a smooth, grooved face, with some collapses (Figure 9a). The surface profile in the collapsing area contained a flat region in the middle of the profile curve. 

Since the lowest depth of the worn surface (300 μm) exceeded the thickness of the surface coating (Figure 9b), it can be concluded that wear damage penetrated the surface coating and inner parts of the GFRP were exposed to wear. The buckypaper coating provided a greater protective function compared to the interlayer embedment sample (Figure 9c) by reducing the breakage of fibers and resin. However, naked glass fibers still remained on the surface (Figure 9d) and broken fiber debris can be observed throughout the collapsed area (Figure 8e), suggesting that the buckypaper coating did not provide a supportive transfer film. The counterpart penetrated the coating and directly imposed shear stress on the glass fiber reinforcement, resulting in friction damage.

**Figure 10:**
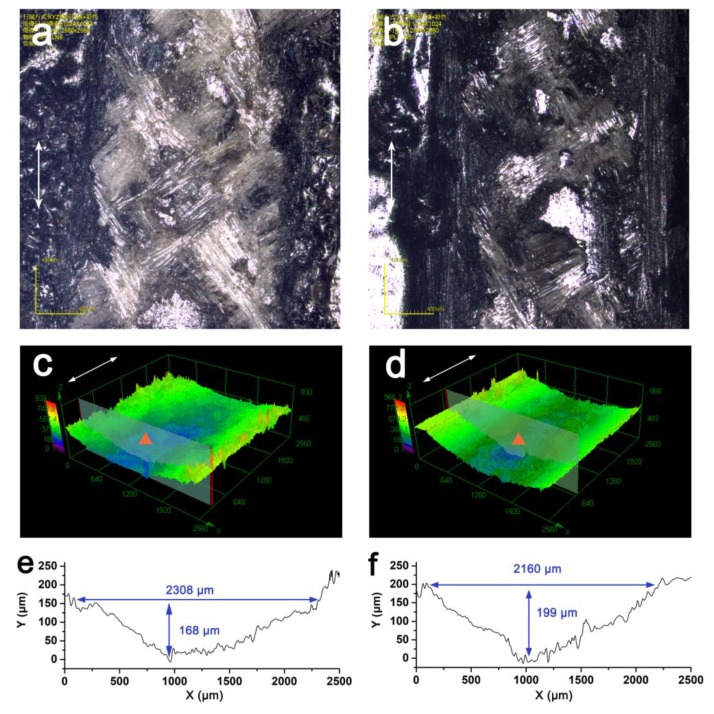
Morphology of worn surface of coating preparation: (**a**,**b**) Optical images of worn surface, (**c**,**d**) 3D surface topography, and (**e**,**f**) surface profiles with C-0.05 g (**a**,**c**,**e**) or C-0.2 g (**b**,**d**,**f**).

From the discussion above, it is essential to supply a supportive transfer film between the composite and the counterpart to promote relative sliding friction and, thus, reduce wear. The protective effect was further examined by decreasing the thickness of the applied coating. After the 20-min reciprocating sliding test, the worn surface of C-0.05 g exhibited a blurred morphology indicating that a large quantity of fibers broke due to plowing and scuffing from the counterpart (Figure 10a). In contrast, increasing the loading to 0.2 g alleviated some of the wear on the surface (Figure 10b), suggesting that a thicker coating maintains a durable transfer film during the sliding abrasion process. Moreover, compared with the topography of the buckypaper coating sample, the worn surface of the coating sample remained relatively smooth with no collapsed area (Figure 10c,d). 

The worn depth of the coating sample increased with thickness of the coating preparation. However, the real worn depth of the protected composite (after subtracting the coating thickness) exhibited the opposite trend (Figure 10e,f). In terms of C-0.4 g, the real worn depths for C-0.05 g and C-0.2 g loading were 89 μm and 28 μm, respectively, indicating the protective effect increased with thickness of the coating. As discussed above, the graphene coating structure became denser as the amount of graphene used in the molding process increased, and can create a durable film between the contact surfaces, thereby improving the wear behavior of the composite.

**Figure 11:**
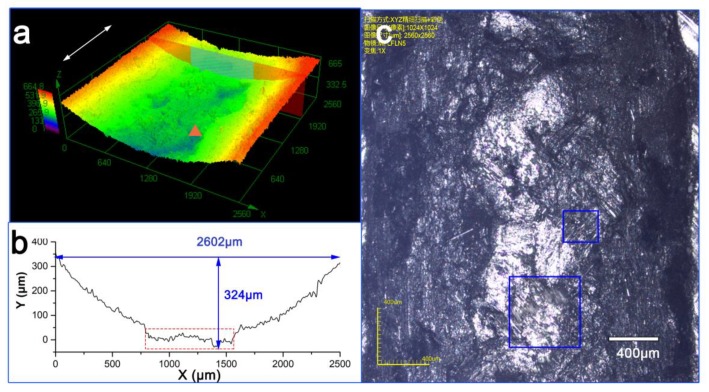
Morphology of worn surface of C-0.4 g after 60-min wear test: (**a**) 3D surface topography, (**b**) surface profile, (**c**) optical image of worn surface.

When the test time was increased to 60 min, the C-0.4 g sample still maintained a smooth, worn surface, except for a small area of scratches (Figure 11a). The worn surface profile exhibited a flat region with some fluctuation in the middle of the profile curve (Figure 11b), corresponding to the region of the graphene coating that was penetrated. After the test, most of the graphene coating remained adhered to the worn surface, while only a small area of fiber breakage and pull out were observed (Figure 11c). This protective effect indicates that the proposed C-0.4 g can provide a durable transfer film to protect against the sliding abrasion. 

**Figure 12:**
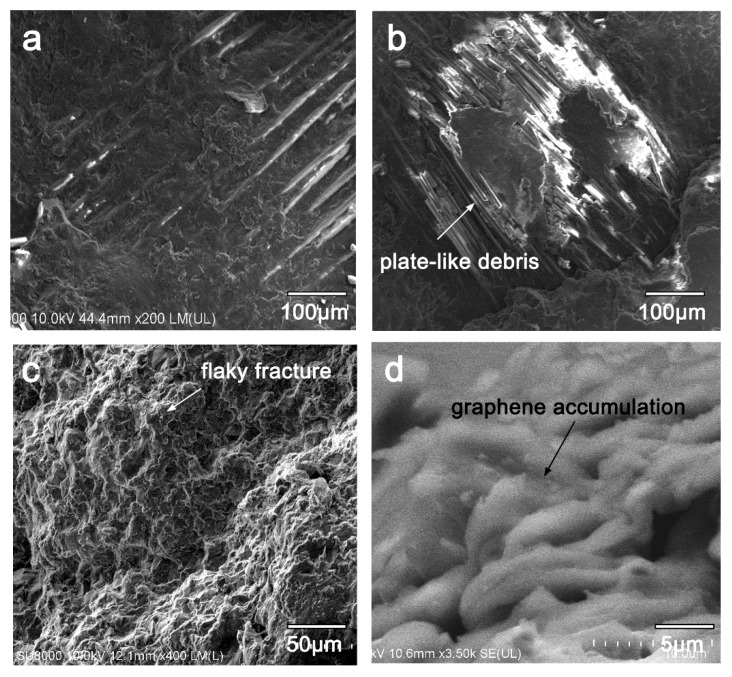
Representative scanning electron micrographs of worn surfaces of C-0.4 g: (**a**,**b**) Enlarged view of worn surface in Sections 1 and 2 (from Figure 11c), (**c**,**d**) lateral observation of the coating preparation.

Details of the worn surface of the coating sample are presented in Figure 12. The remaining resin covered the fibers, protecting them against reciprocating abrasion (Figure 12a). Continuous sliding contact eventually broke down some fibers (Figure 11b). Residual plate-like debris originating from the graphene layer served as the abrasive medium, whereas harder glass fiber debris was avoided, which can reduce stress concentrations created by the rubbing counterpart [18].

A lateral view of the worn integrated coating shows that fractures exhibited a flaky shape (Figure 12c,d), contributing to the accumulation of closed-packed graphene structures in the prepared coating. When the counterpart applied pressure to the integrated coating, the graphene coating was further compressed and formed an even denser transfer film between the counterpart and the composite. Owing to the weak fracture energy of the resin matrix, the resin matrix alone cannot withstand compressive stress induced by the counterpart. Therefore, excess stress must be absorbed by the brittle glass fibers of the raw GFRP, leading to extensive fiber breakage [27]. Conversely, with the graphene coating, a high-quality transfer film formed and most of the compressive stress was dissipated throughout the resin matrix. The increased contact area also prevented plowing and scuffing and, therefore, prevented catastrophic fiber breakage. 

### 3.3. Tribological Discussions

**Figure 13:**
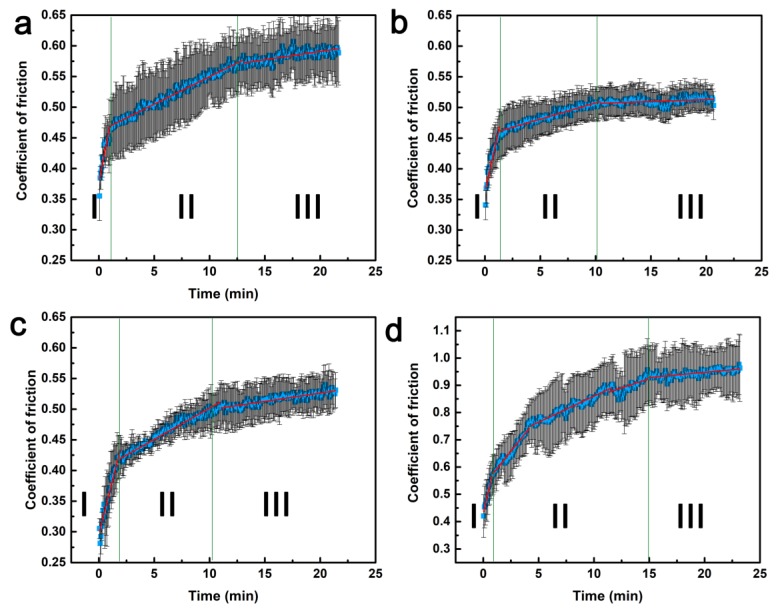
Variation of COF of prepared composites: (**a**) Raw GFRP, (**b**) I-0.4 g, (**c**) B-0.4 g, (**d**) C-0.4 g.

**Figure 14:**
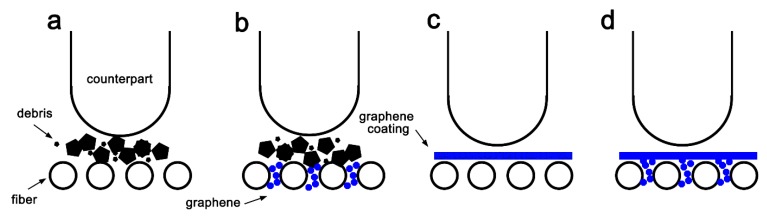
Conceptual model of wear generation: (**a**) Raw GFRP, (**b**) interlayer graphene embedment, (**c**) buckypaper coating, (**d**) graphene coating preparation.

In Figure 13, evolution of COF can be divided into three stages: Initial run-in period, slow growth period, and steady-state period [25]. Each stage is divided using a segmentation fitting method, and the slope of the fitting line represents the growth rate of COF in this stage. For raw GFRP (Figure 13a), the COF started out with a relatively low value of 0.35 and rapidly increased as the production of debris began to hinder the movement of the counterpart. The run-in period transformed into the slow growth period once the transfer film formed and was compressed by the counterpart. The resin layer promoted sliding movement, decreasing the growth rate of the COF [28]. However, as more broken glass fibers are crushed by the counterpart and gradually accumulate in the transfer film, fragments of broken glass fibers can serve as abrasive particles, which hinder sliding movement. This led to abrasive wear as more material was removed by plowing and scuffing (Figure 14a). The sliding movement ejected debris out of the contact area and the sliding counterpart gradually pulverized the broken fibers, thereby reducing the obstructive effect. Finally, after a certain amount of time, the COF reached the steady state [24].

The COF of the interlayer embedment sample followed a similar pattern (Figure 13b), but stabilized at about 0.5, which was smaller than the steady-state COF of raw GFRP. The pinning effect of graphene in the composite improved the bonding strength between the fibers and matrix, thereby decreasing the amount of fiber debris (Figure 13b) [29]. Consequently, the sliding abrasive movement became smooth and gentle, and the length of the run-in period and slow growth period were reduced. 

The buckypaper coating produced a COF curve similar to that of raw GFRP and interlayer embedment (Figure 13c). The self-lubricating characteristics of the graphene surface coating minimized contact between the friction pair and decreased the COF (Figure 14c) [30]. However, observation of the worn surface suggests debris was not widely distributed across the worn surface and the transfer film could not withstand the contact pressure. Therefore, the counterpart penetrated the transfer film and was in direct contact with the GFRP composite, leading to friction behavior similar to GFRP. 

Compared to the buckypaper coating, the graphene coating in this paper led to continuous growth of the COF, which gradually increased up to 0.9 (Figure 13d), indicating that a high friction load was applied to the sample. After a short running-in stage, the growth rate became gradually decreased, but still contributed to a large margin until the COF stabilized at around15 min. In this coating preparation, graphene can be assembled layer-by-layer and compacted to form a dense transfer film between the composite and the counterpart. The formation of a high-quality transfer film improved the antiwear properties of the composite. Owing to the durable transfer film, resin in the coating wore away more easily due to sliding abrasion, leaving plate-like debris on the transfer film. Therefore, the transfer film became more ductile, and the counterpart began to sink into the transfer film. As a result, the friction torque and, as a consequence, the COF gradually increased. When the transfer film was peeled off by the counterpart, the COF reached a steady state of around 0.9. In addition to the high-quality transfer film, the graphene coating produced a much greater pinning effect, compared to the buckypaper coating, by infusing graphene into the fibrous medium, thus further improving antiwear performance (Figure 14d).

## 4. Conclusions

The results of this investigation demonstrate a feasible approach for improving the wear resistance of GFRP. Graphene was accumulated on the GFRP surface via filtration mechanisms using a modified percolating-assisted resin film infusion method. The obtained graphene coating exhibited superior antiwear resistance compared to interlayer embedment and the buckypaper coating. As the graphene content was increased in the coating preparation, the protective properties improved. The proposed coating sustained loading of 0.4 g while completely protecting the inner composite during the first 20-min sliding abrasion test, and continued to be protective for the following 40-min test. By analyzing the morphology of the worn surface and the tribological process, the wear mechanisms were revealed. Compared with other samples, the coating preparation can simultaneously provide a durable transfer film while promoting the pinning effect. Micrographs of the worn surface show that the layer of assembled graphene in the graphene coating was compacted by the counterpart to form a dense transfer film, which improved the wear behavior of the composite by preventing direct contact between surfaces of the composite and the rubbing counterpart.
